# Adaptive Data Aggregation and Compression to Improve Energy Utilization in Solar-Powered Wireless Sensor Networks

**DOI:** 10.3390/s17061226

**Published:** 2017-05-27

**Authors:** Ikjune Yoon, Hyeok Kim, Dong Kun Noh

**Affiliations:** 1 Department of Smart Systems Software, Soongsil University, Seoul 06978, Korea; ijyoon@ssu.ac.kr; 2 Department of Software Convergence, Soongsil University, Seoul 06978, Korea; hk@ssu.ac.kr

**Keywords:** wireless sensor network, energy-harvesting, solar-powered, aggregation, compression

## Abstract

A node in a solar-powered wireless sensor network (WSN) collects energy when the sun shines and stores it in a battery or capacitor for use when no solar power is available, in particular at night. In our scheme, each tiny node in a WSN periodically determines its energy budget, which takes into account its residual energy, and its likely acquisition and consumption. If it expects to acquire more energy than it can store, the data which has it has sensed is aggregated with data from other nodes, compressed, and transmitted. Otherwise, the node continues to sense data, but turns off its wireless communication to reduce energy consumption. We compared several schemes by simulation. Our scheme reduced the number of nodes forced to black out due to lack of energy so that more data arrives at the sink node.

## 1. Introduction

Sensor networks are used to obtain environmental information such as temperature, humidity, and pressure. Wireless sensor networks (WSNs) are suitable for less accessible areas such as extended open spaces, battlefields, or deep water. The nodes in WSNs are usually powered by batteries, and are often simply discarded when their batteries are exhausted. This has motivated the introduction of techniques for reducing energy consumption to prolong network lifetime [1,2], using energy-harvesting nodes [3] that obtain energy from various sources such as the sun [4,5,6], vibrations [7,8], wind [9,10], and temperature differences [11]. Solar energy is the most popular because of its high areal density (about 15 $\mathrm{mW}/{\mathrm{cm}}^{2}$) [12], even though the availability of solar power depends on the time of day and the weather. Recent work on energy-harvesting WSNs has largely been concerned with using energy efficiently, so that a node can survive as long as possible [13,14,15].

Each node in a WSN transmits its own data and forwards data from other nodes towards a sink node. Thus, nodes near the sink node consume more energy, which may cause them to stop operating temporarily, or black out, more frequently than other nodes. This problem can be addressed by data aggregation schemes [16,17], in which relay nodes combine their own data received from other nodes. This can improve the energy efficiency of data transmission, but it increases the transmission delay because relay nodes need to wait for the appropriate data to aggregate with their own. Thus, aggregation schemes are only used in networks [18] in which the time at which data arrives is not critical.

Another tool for reducing energy consumption is data compression. This requires considerable processing time, and hence energy, but if nodes far from the sink which are relatively rich in energy compress their own data, nodes closer to the sink will use less energy in relaying that data, and then these nodes also save energy by not compressing their own data. Thus, energy consumption becomes more balanced across the network. The scope for an advantageous trade-off depends on the size of WSN.

In conventional aggregation and compression schemes, cluster heads generally discard redundant data and compress it using lossy compression algorithms to minimize the amount of transmission data. The schemes have also studied how to improve the accuracy of recovered data. Since most of these schemes are designed for a cluster topology, they are not suitable for a flat topology. Moreover, they can not be applied in the case that all of the data should be collected because they have to discard redundant data to reduce the amount of data transmitted. In addition, they are not appropriate for solar-powered nodes because they are designed to minimize energy consumption in battery-powered WSNs.

In this paper, we address these issues with an adaptive data aggregation and compression schemes for solar-powered WSNs, in which all data should be transmitted to the sink node.

It increases the amount of data that reaches the sink node safely by arranging for data to be compressed when energy is available. In our scheme, each node periodically estimates the state of its battery and forecasts the amount of solar energy that it will acquire during the subsequent period. If it expects to acquire more energy than it can store, it transmits the data that it has aggregated. Conversely, if it expects to exhaust its energy during the subsequent period, then it enters sleep mode, turns off its wireless module, and continues sensing only. This increases the amount of data collected by reducing the number of nodes that black out. The proposed scheme assumes that it operates in a flat topology, but it can be easily adopted to a cluster topology and be applied to various in-node compression algorithms.

The rest of this paper is organized as follows. In Section 2, we describe the background to this research and review related work. In Section 3, we introduce our scheme for data aggregation and compression, and describe how a node determines its mode, and whether to transmit data. In Section 4, we present experimental results and assess the performance of our scheme. Section 5 concludes the paper.

## 2. Related Work

In this section, we will review methods for data aggregation, compression and energy utilization.

### 2.1. Data Aggregation in WSNs

Data aggregation has been shown to be effective in reducing energy consumption in WSNs [17] by reducing the amount of data that has to be transmitted from one node to another [19].

Clustering is one method of data aggregation, in which each node only transmits data to a node designated as a cluster head. Each cluster head aggregates the data received from the members of its cluster, and transmits it towards the sink node, either directly or through other cluster heads. Heinzelman [20] introduced a low-energy adaptive clustering hierarchy (LEACH), which equalizes energy usage across a WSN by arranging for nodes to take turns as cluster heads. Voigt et al. [21] extended LEACH to WSNs with a mixture of solar-powered and battery-only nodes. In the solar-aware LEACH (sLEACH) algorithm, solar-powered nodes are preferentially chosen to undertake the extra transmissions required from cluster heads. Chatterjea and Havinga [22] proposed a data aggregation scheme which combines clustering with directed diffusion in which different types of data are recognized and treated differently during transmission [23]. Ghaffariyan [24] explained the differences between three clustering schemes, LEACH, a hybrid, energy-efficient, distributed clustering (HEED), and a distributed weight-based energy efficient hierarchical clustering (DWEHC), and presented the effect of each on predictive data aggregation and data summarization mechanisms. Boyd et al. [25] applied a gossip algorithm to data aggregation, in which the data from some nodes is not transmitted but inferred. In most of the aggregation schemes studied so far, clustering is applied and nodes remove unnecessary data and transfer only some samples, so that the sink node can estimate the original data. However, in this paper, we propose an aggregation policy that can be applied when all data should be transmitted in a flat topology without cluster, and an energy management policy that can apply many existing aggregation schemes to our scheme.

### 2.2. Data Compression in WSNs

Data compression is another method of reducing the energy required for data transmission. However, the data compression algorithms used in general-purpose computers are not suitable for the limited hardware in wireless sensor nodes, and low-overhead compression algorithms have therefore been introduced [26,27].

Sadler and Martonosi [28] proposed the sensor Lempel–Ziv–Welch(S-LZW) algorithm, which was the representative directory-based LZW lossless compression algorithm. Petrovic et al. [29] proposed a data funneling scheme, in which the setup and control packets used for routing are compressed using a lossless ordering method, so as to reduce transmission energy. Arici et al. [30] proposed the a pipelined in-network compression scheme (PINCO), which reduces the redundancy that is usually present in data collected from several nodes, reducing the need for transmission and hence energy consumption. In this scheme, aggregated data is efficiently recompressed without decompressing. Kasirajan et al. [31] proposed a lossy compression technique that uses the principle of an adaptive differential pulse-code modulation (ADPCM) in a clustering environment to improve the energy utilization. In the scheme, a source node approximates the sample value and only transmits its quantized estimate instead of the actual data sample. In our scheme, nodes aggregate a specific amount of sensed data to achieve the maximum compression ratio, and compress and transmit the data. In this case, we use the S-LZW scheme as the compression method, which is a lossless compression algorithm widely used in WSNs. The other compression schemes for WSNs are designed to depend on aggregation schemes, but our scheme can apply S-LZW as well as many other compression algorithms.

### 2.3. Energy Utilization in WSNs

Several methods of increasing energy utilization have been studied to prolong the lifetime of WSNs except for the aforementioned data aggregation and compression schemes. Many researchers have proposed schemes to increase energy efficiency by adjusting the transmission power and determining the route accordingly. Zanella et al. mathematically analyzed the effect of routing on interference [32]. They also presented a mathematical model for selecting relay nodes in a two-hop multi-user system [33]. They also have investigated the effects of signal level-based power control (SLPC) by considering channel gain, and transmit and receive power levels in ad hoc wireless networks [34]. Palombara et al. [35] developed a framework, FEP-optimal power allocation, which assessing the frame error probability (FEP) to analyze relay-assisted diversity communications. Unlike the schemes mentioned above, we improve energy utilization by using the harvested solar energy for the data aggregation and compression within the energy depletion or excess.

In energy-harvesting WSNs, many authors [12,36,37,38] have contributed models and techniques for estimating the availability of environmental energy and enabling sensor nodes to make the best use of it. Cammarano et al. [39] devised an accurate solar energy prediction scheme. It can predict accurate solar energy changes by time and weather condition using long-term and short-term predictions. Kansal et al. [37] introduced an energy model for solar-powered nodes, which determines the bounds of energy that allow a node to survive indefinitely. Yang et al. [38] proposed an energy management scheme, which considers consuming power $P_{\mathrm{sys}}$ and harvesting power $P_{\mathrm{solar}}$ of a solar-powered sensor node. Roundy et al. [12,36] proposed methods of estimating the amount of energy that can be obtained from the energy sources shown in Table 1. Noh et al. [40] proposed a routing scheme to determine the transmission path for three cases, considering the harvested energy in solar-powered WSNs. Kang et al. [41] proposed a scheme to reduce data transmission delay by determining whether to compress data according to the harvested energy in solar-powered WSNs. Our scheme manages the energy required for data aggregation, compression, and transmission using the aforementioned solar energy prediction [39] and consumption model [37] for the solar-powered nodes.

## 3. Adaptive Aggregation and Compression Scheme

**Figure 1:**
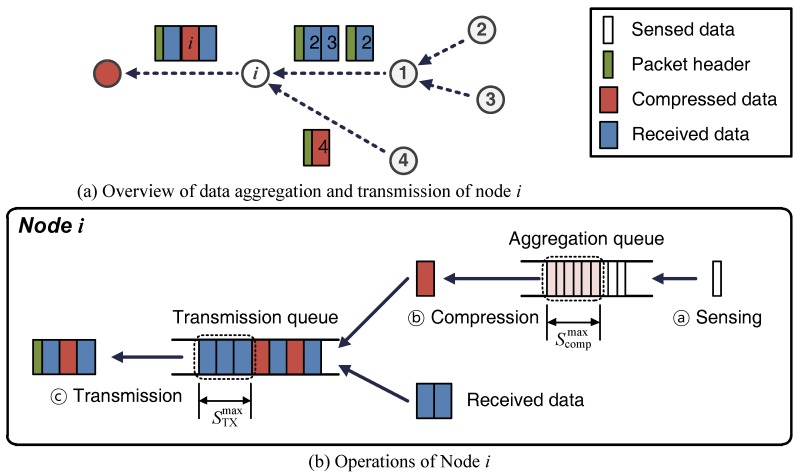
Overview of data transmissions and node operations.

Our scheme, shown in Figure 1, is designed for a delay-tolerant network [18] of nodes periodically collecting data from their environments.

### 3.1. Sensor Node Operations

Each node senses its environment, compresses the resulting data, and transmits it toward the sink node as shown in Figure 1b. Each node also reviews its status at the end of a fixed period of time, called a round, and determines the mode in which it will operate during the next round. Here are some more details of these operations:

#### Sensing

Data is collected at the interval of $p_{\mathrm{sense}}$, which is the sensing period.

#### Compression

The data is compressed when enough has been gathered to allow the maximum compression to be achieved. The compressed data and data received from other nodes are then queued for transmission.

#### Transmission

At intervals of $p_{\mathrm{Tx}}$, a node determines whether to transmit the data in its transmission queue. If the node expects to receive more energy than it can store during the next $p_{\mathrm{Tx}}$, then the data is transmitted.

#### Mode selection

At the end of each round, of length $p_{\mathrm{round}}$, a node selects its mode by estimating whether the energy in its battery would run out if it were to continue communicating with other nodes. The way in which this estimate is made is described in the next section.

- *Normal mode* is selected when there is sufficient energy to continue sensing, compression, and transmission during the next round.
- *Sleep mode* is selected if the residual energy would otherwise run out during the next round. In this mode, a node only performs sensing and compression. It turns off its wireless module, and thus any data sent to it during the subsequent period is lost . To avoid this happening, nodes select their modes before the routing process, which excludes nodes in sleep mode.

### 3.2. Mode Selection

To determine the amount of data that a node has to transmit in one round, we need to work out the time it requires to compress the data it has gathered so far and put it into the transmission queue. If a node obtains $S_{\mathrm{sense}}$ bits of data during each period $p_{\mathrm{sense}}$, and aggregates it up to $S_{\mathrm{comp}}^{\max}$ bits and compresses it, then the length of $p_{\mathrm{comp}}$ required for data compression can be determined as follows:

(1) $$p_{\mathrm{comp}}=\frac{S_{\mathrm{comp}}^{\max}}{S_{\mathrm{sense}}}p_{\mathrm{sense}}.$$

**Figure 2:**
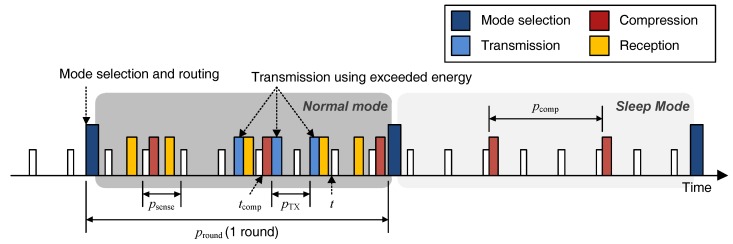
Process of the proposed scheme.

Therefore, as shown in Figure 2, if the current time is *t* and the most recent data compression was completed at $t_{\mathrm{comp}}$, the number of times that the compressed data have to be added to the transmission queue during the next round can be expressed as follows:

(2) $$k\left(t,p_{\mathrm{round}}\right)=\left\lfloor \frac{p_{\mathrm{round}}+\left(t-t_{\mathrm{comp}}\right)}{p_{\mathrm{comp}}}\right\rfloor .$$

Given $R_{\mathrm{comp}}\left(S\right)$ denotes the compression ratio when compressing *S* bits data, $S_{\mathrm{comp}}^{\max}$ is compressed to $S_{\mathrm{comp}}^{\max}/R_{\mathrm{comp}}\left(S_{\mathrm{comp}}^{\max}\right)$. Thus, the amount of data to be put on to the transmission queue during the next round $S_{\mathrm{round}}\left(t,p_{\mathrm{round}}\right)$ can be represented as follows:

(3) $$S_{\mathrm{round}}\left(t,p_{\mathrm{round}}\right)=k\left(t,p_{\mathrm{round}}\right)\frac{S_{\mathrm{comp}}^{\max}}{R_{\mathrm{comp}}\left(S_{\mathrm{comp}}^{\max}\right)}+S_{\mathrm{relay}}\left(t,p_{\mathrm{round}}\right),$$

where $S_{\mathrm{relay}}\left(t,p_{\mathrm{round}}\right)$ is the amount of data received from other nodes during the next round. Data is transmitted in packets of size $S_{\mathrm{Tx}}^{\max}$, which have headers. The total number of bits of packets that need to be transmitted to forward the data accumulated during a round can then be calculated as follows:

(4) $$S_{\mathrm{round}}^{\mathrm{packet}}\left(t,p_{\mathrm{round}}\right)=\left\lceil \frac{S_{\mathrm{round}}\left(t,p_{\mathrm{round}}\right)}{S_{\mathrm{Tx}}^{\max}}\right\rceil S_{\mathrm{header}}+S_{\mathrm{round}}\left(t,p_{\mathrm{round}}\right).$$

We now determine the amount of data that can be transmitted using the solar energy, $E_{\mathrm{Tx}}$ acquired during a round, using the energy consumption model of Melodia et al. [43]:

(5) $$E_{\mathrm{Tx}}=S\beta d^{\alpha },$$

where *S* is the number of bits of data to be transmitted, *d* is the transmission distance in meters, and α is the path loss exponent (2≤α≤5); The constant β ($J/(\mathrm{bits}\cdot m^{\alpha })$) is determined by the design of the node. From Equation (5), we can determine the amount of data ${\hat{S}}_{h}$ that can be transmitted by the energy harvested during a round:

(6) $${\hat{S}}_{h}\left(t,p_{\mathrm{round}}\right)=\frac{{\hat{E}}_{h}\left(t,p_{\mathrm{round}}\right)-{\hat{E}}_{c}\left(t,p_{\mathrm{round}}\right)-E_{\mathrm{comp}}\left(S_{\mathrm{round}}\left(t,p_{\mathrm{round}}\right)\right)}{\beta d^{\alpha }},$$

where ${\hat{E}}_{h}\left(t,p_{\mathrm{round}}\right)$ is the amount of energy expected to be acquired during the next round, which can be estimated in several ways [39,44,45,46]. ${\hat{E}}_{c}\left(t,p_{\mathrm{round}}\right)$ is the estimated energy consumption during $p_{\mathrm{round}}$, which includes idle and receiving energy, and $E_{\mathrm{comp}}\left(S_{\mathrm{round}}\left(t,p_{\mathrm{round}}\right)\right)$ is the amount energy consumed in compressing the $S_{\mathrm{round}}\left(t,p_{\mathrm{round}}\right)$ bits of data.

By comparing the values $S_{\mathrm{round}}^{\mathrm{packet}}\left(t,p_{\mathrm{round}}\right)$, obtained from Equation (4), with the value of ${\hat{S}}_{h}$, obtained from Equation (6), a node can determine whether it is likely to be able to transmit all its data during the current round using solar energy. If $S_{\mathrm{round}}^{\mathrm{packet}}\left(t,p_{\mathrm{round}}\right)>{\hat{S}}_{h}$, then it is likely that some data will not be transmitted and will accumulate. To avoid this, the node enters sleep mode. Conversely, if $S_{\mathrm{round}}^{\mathrm{packet}}\left(t,p_{\mathrm{round}}\right)\leq {\hat{S}}_{h}$, the node continues normal operation and transmits its data.

### 3.3. Choosing Whether to Transmit Data

If a node expects to acquire more energy than it can store, it uses that energy to transmit data. We now show how it can be determined whether there is likely to be enough stored energy available during $p_{\mathrm{Tx}}$.

If the energy available to a node at time *t* is $E_{r}\left(t\right)$, the residual energy after the transmission period $p_{\mathrm{Tx}}$ can be estimated as follows:

(7) $${\hat{E}}_{r}\left(t,p_{\mathrm{Tx}}\right)=E_{r}\left(t\right)+{\hat{E}}_{h}\left(t,p_{\mathrm{Tx}}\right)-{\hat{E}}_{c}\left(t,p_{\mathrm{Tx}}\right)-E_{\mathrm{comp}}\left(S_{\mathrm{round}}\left(t,p_{\mathrm{round}}\right)\right).$$

If the capacity of the node’s battery is *C*, the excess energy likely to be acquired during the round is

(8) $${\hat{E}}_{\mathrm{excess}}\left(t,p_{\mathrm{Tx}}\right)=C-\left(E_{r}\left(t\right)+{\hat{E}}_{h}\left(t,p_{\mathrm{Tx}}\right)-{\hat{E}}_{c}\left(t,p_{\mathrm{Tx}}\right)-E_{\mathrm{comp}}\left(S_{\mathrm{round}}\left(t,p_{\mathrm{round}}\right)\right)\right).$$

If ${\hat{E}}_{\mathrm{excess}}\left(t,p_{\mathrm{Tx}}\right)>0$, then the node transmits data. The number of bits of packets ${\hat{S}}_{\mathrm{avail}}^{\mathrm{packet}}\left(t,p_{\mathrm{Tx}}\right)$, which can be transmitted using ${\hat{E}}_{\mathrm{excess}}\left(t,p_{\mathrm{Tx}}\right),$ can be derived using Equation (5):

(9) $${\hat{S}}_{\mathrm{avail}}^{\mathrm{packet}}\left(t,p_{\mathrm{Tx}}\right)=\frac{{\hat{E}}_{\mathrm{excess}}\left(t,p_{\mathrm{Tx}}\right)}{\beta d_{i}^{\alpha }}.$$

The actual number of bits of data excluding the packet header ${\hat{S}}_{\mathrm{avail}}\left(t,p_{\mathrm{Tx}}\right)$ is

(10) $${\hat{S}}_{\mathrm{avail}}\left(t,p_{\mathrm{Tx}}\right)={\hat{S}}_{\mathrm{avail}}^{\mathrm{packet}}\left(t,p_{\mathrm{Tx}}\right)-\left\lceil \frac{{\hat{S}}_{\mathrm{avail}}^{\mathrm{packet}}\left(t,p_{\mathrm{Tx}}\right)}{S_{\mathrm{Tx}}^{\max}}\right\rceil S_{\mathrm{header}}.$$

**Figure 3:**
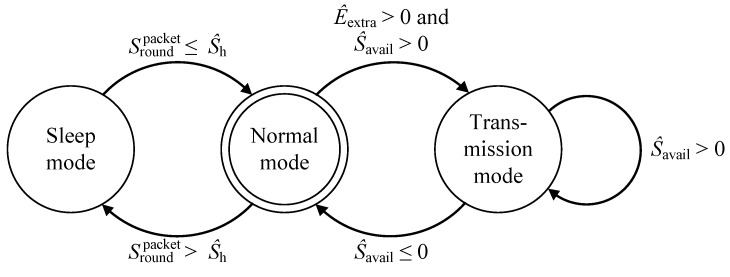
State transition diagram of node operations.

Therefore, if ${\hat{E}}_{\mathrm{excess}}\left(t,p_{\mathrm{Tx}}\right)>0$, then the node transmits ${\hat{S}}_{\mathrm{avail}}\left(t,p_{\mathrm{Tx}}\right)$ bits of data. Figure 3 shows the transitions between sleep, normal, and transmission mode.

This scheme allows nodes to obtain data during periods when solar energy is not available.

## 4. Performance Evaluation

### 4.1. Simulation

We used SolarCastalia [47] to compare the performance of our scheme with others: (1) no data aggregation and compression (Naive); (2) aggregating data at regular intervals (Aggr-time); (3) aggregating a regular amount of data (Aggr-size); (4) compressing aggregated data at regular intervals (Comp-time); and (5) compressing a regular amount of aggregated data (Comp-size). We used the average residual energy in a node, the number of black-out nodes, and the amount of data arriving at the sink node as measures of performance. The simulated WSN consisted of 100 energy-harvesting nodes and one sink node, placed at random positions. The amount of energy harvested by the nodes was modeled by measured data [47], and S-LZW [28] was used as the compression algorithm. At the beginning of every round, each node determines its mode using the algorithm described in Section 3.2, and finds a routes to the sink node using the minimum depth tree (MDT) algorithm. After sensing, each node performs the aggregation, compression and transmission operations according to the schemes described above. Table 2 contains the important parameters used in our simulation.

### 4.2. Simulation Results

#### 4.2.1. Residual Energy and Blackout Nodes

**Figure 4:**
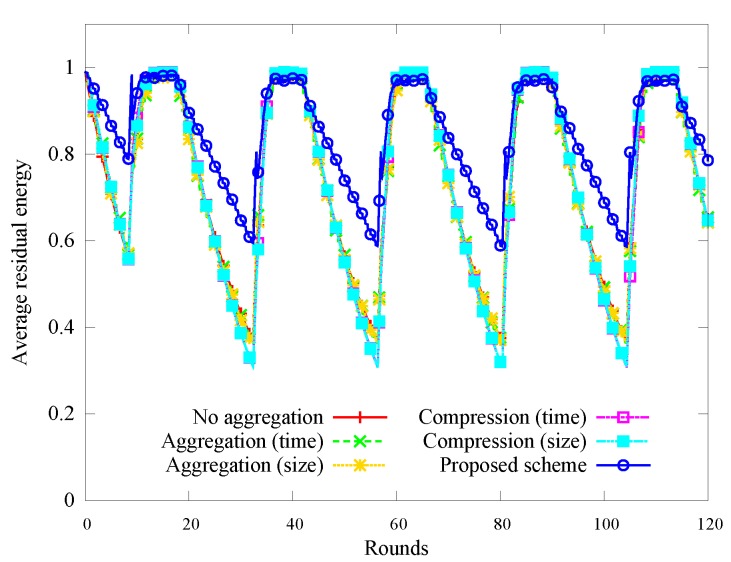
Change in the average amount of residual energy.

Figure 4 shows the change in the average amount of residual energy over time, which follows a diurnal cycle. The overnight loss in energy is less with our scheme, in which nodes enter sleep mode based on energy prediction.

**Figure 5:**
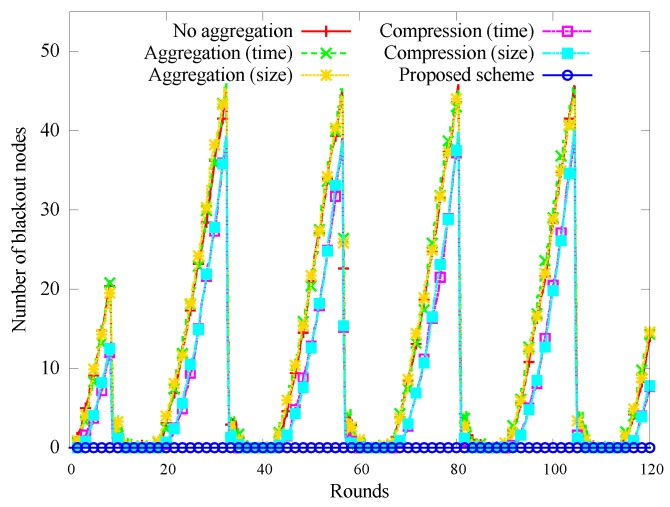
Change in the number of black-out nodes.

Figure 5 shows how the number of black-out nodes changes over time. In the Naive, Aggr-time, and Aggr-size schemes, the number of blackout nodes increased more rapidly. We attribute this to the increased amount of data that had to be transferred by intermediate nodes. This was lower in schemes using compression, which cause fewer nodes to black out. Our scheme puts nodes to sleep at night, and very few blackouts occur.

**Figure 6:**
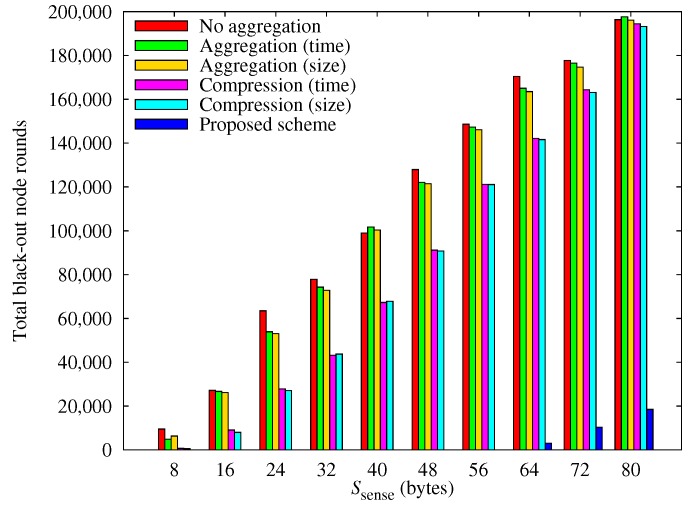
Number of cumulative black-out nodes.

Figure 6 shows the total number of black-out node-rounds (i.e., a total that is incremented every time any node is blacked out for a round) that have occurred by the 8000th round varies with the value of $S_{\mathrm{sense}}$. With our scheme, the number of blacked-out node-rounds is negligible until $S_{\mathrm{sense}}$ reaches 64 bytes.

#### 4.2.2. Amount of Data Arriving at the Sink Node

**Figure 7:**
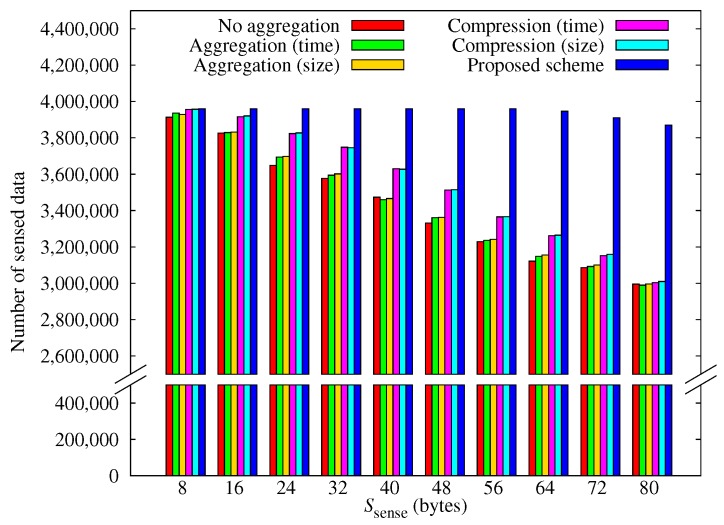
Comparison of the total amount of sensed data.

Figure 7 shows how the amount of data sensed by all nodes varies with $S_{\mathrm{sense}}$. These results correlate with the number of black-out nodes, shown in Figure 6, because blacked-out nodes collect no data.

**Figure 8:**
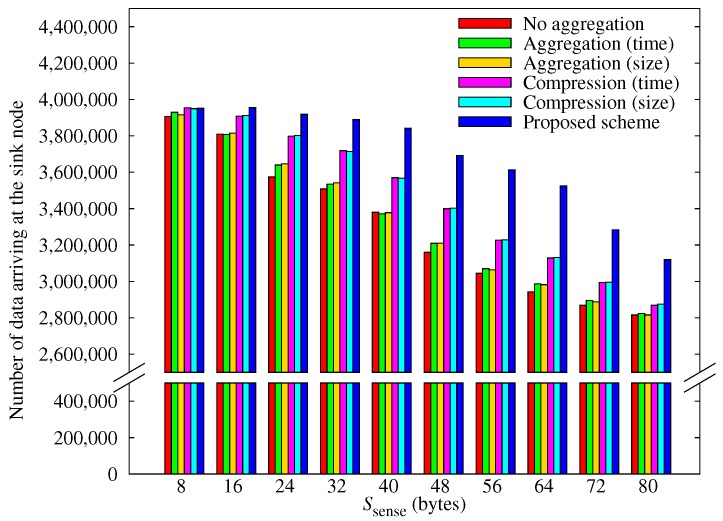
Comparison of the amount of data obtained according to the sensing data size.

Figure 8 shows how the amount of data arriving at the sink node varies with $S_{\mathrm{sense}}$. These results are similar to those shown in Figure 6, except that the proportion of the sensed data that our scheme delivers to the sink node gradually falls. We can attribute this effect to energy constraints on the capacity of the network to relay data. The other schemes are not affected simply because they do not produce sufficient data for transmission to become a problem due to many black-out nodes.

**Figure 9:**
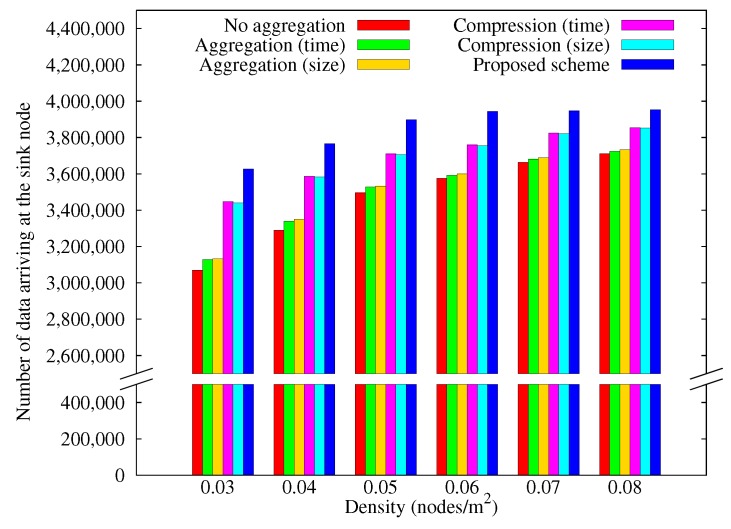
Change in the amount of data arriving at the sink node with node density.

Figure 9 shows how the amount of data arriving at the sink node varies with nodes density. As the density increases, it becomes easier to find alternative paths when some nodes black out, so move data that arrives at the sink node increased as the density increases. Our scheme continues to outperform the other schemes as density is increased.

**Figure 10:**
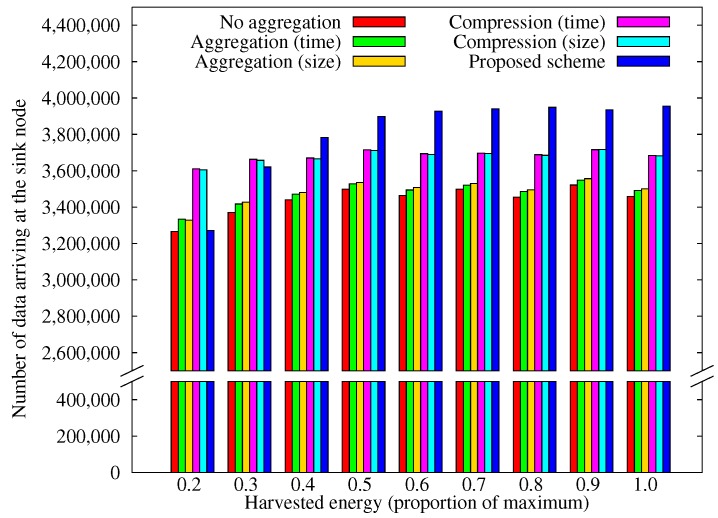
Comparison of the amount of data obtained according to the solar energy.

Figure 10 shows how the amount of data arriving at the sink node varies with the amount of solar energy that the nodes can acquire. The energy available is reduced by applying a factor to the energy model [47]. As the available energy declines, the relative performance of our scheme decreased noticeably. This is plausible because our scheme only allows nodes to transmit data when they expect to have excess energy, and fewer nodes find themselves in this situation when the supply of energy is reduced.

**Figure 11:**
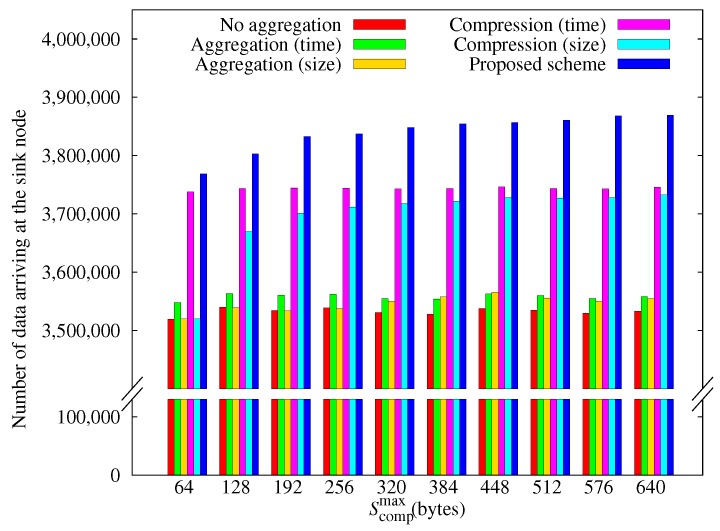
Comparison of the amount of data obtained according to the maximum aggregation size.

Figure 11 shows how the amount of data arriving at the sink node varies with $S_{\mathrm{comp}}^{\max}$. As $S_{\mathrm{comp}}^{\max}$ decreases, the performances of the comp-size scheme and the proposed scheme decrease, whereas the others are not affected. We can attribute this effect to the low compression ratio of small size data. Since the amount of compressed data increases as the compression ratio becomes smaller, the node must transmit more data.

**Figure 12:**
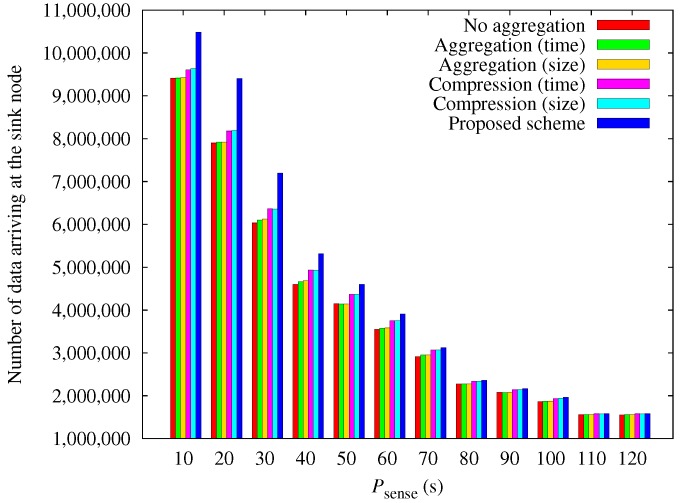
Comparison of the amount of data obtained according to the sensing period.

Figure 12 shows the comparison of the amount of obtained data according to the sensing period. As $p_{\mathrm{sense}}$ increases, less data is transmitted. It makes the number of black-out nodes reduced, and all schemes show similar performances. Conversely, as $p_{\mathrm{sense}}$ decreases, nodes must transmit more data because this increases the amount of sensed data. This increases the amount of energy consumed. The proposed scheme outperforms the other schemes as $p_{\mathrm{sense}}$ is decreased because of the energy depletion of other schemes.

**Figure 13:**
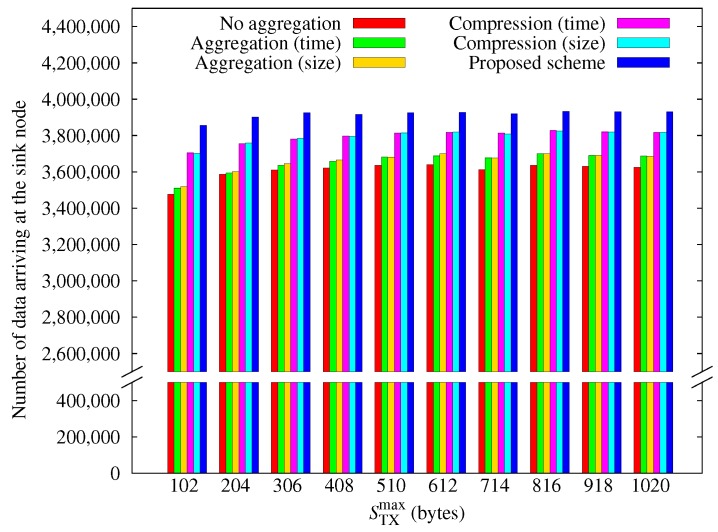
Comparison of the amount of data obtained according to the maximum transmission size.

Figure 13 shows how the amount of data arriving at the sink node varies with $S_{\mathrm{Tx}}^{\max}$. Note that the number of data obtained is decreased when $S_{\mathrm{Tx}}^{\max}$ is smaller than 510 bytes. The reason is that a packet can contain smaller data because the ratio of the header becomes larger and the ratio of the data becomes smaller. This leads to decreasing the number of data obtained. Conversely, if $S_{\mathrm{Tx}}^{\max}$ becomes large, a node can send large packets at a time, reducing the overhead of packet headers. However, the larger the packet size, the greater the risk of packet loss. In other experiments, we used the maximum size of 102 bytes for the 802.15.4 protocol, which is mainly used in WSNs.

**Figure 14:**
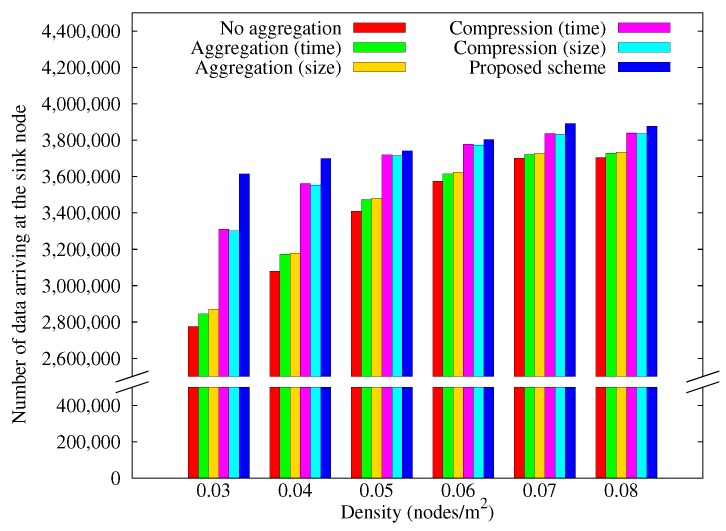
Change in the amount of data arriving at the sink node with node density (greedy perimeter stateless routing (GPSR)).

Figure 14 shows how the amount of data arriving at the sink node varies with node density when we applied greedy perimeter stateless routing (GPSR) [48] as the routing algorithm. This result is similar to Figure 9 when using MDT. However, when using GPSR, somewhat less data have been collected at the sink node because more routing information have to be added to a packet than MDT. As the density decreases, the performance of the schemes using no compression gets worse because packets consume energy in more relay nodes. As the density increases, the energy consumption of the relay nodes is reduced due to the shorter transmission route. Moreover, as the route is updated with every transmission, it becomes easier to find alternative paths to the sink node. As a result, we can see that the deviation of GPSR becomes smaller than MDT. However, the proposed scheme still outperforms the other schemes regardless of the routing algorithms.

## 5. Conclusions

We have proposed a new adaptive aggregation and compression scheme for solar-powered WSNs. In this scheme, a node continually aggregates and compresses sensed data, but only transmits it when it expects to receive more energy than it can store. When there is little or no solar energy available, in particular at night, the nodes stop transmitting and perform nothing but sensing. This scheme reduces the number of nodes that black out and thus allows more data to be obtained. A difficulty with the current scheme is that the network workload can peak when data acquired during the night is transmitted the following morning. Some data may then be lost by forwarding nodes that have insufficient energy. We plan to reduce peaks in the network load by predicting the pattern of energy acquisition and consumption more precisely.

## Figures and Tables

**Table 1:** Power density of environmental energy [12,36,42].

Harvesting Technology	Power Density
Solar cells (outdoors at noon)	15 $\mathrm{mW}/{\mathrm{cm}}^{2}$
Piezoelectric (shoe inserts)	330 $\mu W/{\mathrm{cm}}^{3}$
Vibration (small microwave oven)	116 $\mu W/{\mathrm{cm}}^{3}$
Thermoelectric (10 ℃ gradient)	40 $\mu W/{\mathrm{cm}}^{2}$
Acoustic noise (100 dB)	960 $\mathrm{nW}/{\mathrm{cm}}^{3}$

**Table 2:** Simulation parameters.

Parameters	Values
Number of nodes	100
Node topology	Random
Routing algorithm	Minimum depth tree (MDT)
Transmission range	10∼20 m
$p_{\mathrm{round}}$	1 h
$p_{\mathrm{Tx}}$	300 s
$p_{\mathrm{sense}}$	60 s
$S_{\mathrm{Tx}}^{\max}$	102 bytes
$S_{\mathrm{round}}$	1024 bytes
$S_{\mathrm{sense}}$	8∼80 bytes
